# The evolution of disease resistance and tolerance in spatially structured populations

**DOI:** 10.1002/ece3.290

**Published:** 2012-07

**Authors:** Felix Horns, Michael E Hood

**Affiliations:** Department of Biology, Amherst CollegeAmherst, Massachusetts 01002

**Keywords:** Costs, defense, host–pathogen interactions, parasitism

## Abstract

The ubiquitous challenge from infectious disease has prompted the evolution of diverse host defenses, which can be divided into two broad classes: resistance (which limits pathogen growth and infection) and tolerance (which does not limit infection, but instead reduces or offsets its negative fitness consequences). Resistance and tolerance may provide equivalent short-term benefits, but have fundamentally different epidemiological consequences and thus exhibit different evolutionary behaviors. We consider the evolution of resistance and tolerance in a spatially structured population using a stochastic simulation model. We show that tolerance can invade a population of susceptible individuals (i.e., neither resistant nor tolerant) with higher cost than resistance, even though they each provide equivalent direct benefits to the host, because tolerant hosts impose higher disease burden upon vulnerable competitors. However, in spatially structured settings, tolerance can invade a population of resistant hosts only with lower cost than resistance due to spatial genetic structure and the higher local incidence of disease around invading tolerant individuals. The evolution of tolerance is therefore constrained by spatial genetic structure in a manner not previously revealed by nonspatially explicit models, suggesting mechanisms that could maintain variation or limit the occurrence of tolerance relative to resistance.

## Introduction

Parasites are ubiquitous and have detrimental effects on their hosts (Price 1980), resulting in strong selection for defensive mechanisms that prevent or mitigate the harm caused by infection. These defenses can be divided into two broad classes: resistance and tolerance. Resistance traits reduce the harm caused by disease by preventing infection or limiting subsequent pathogen growth and development within the host through avoidance or clearance of infection. Tolerance traits do not inhibit infection, but instead reduce or offset its negative fitness consequences (Roy and Kirchner 2000; Miller et al. 2005) by reducing the additional mortality due to infection or restoring the reproductive ability (fecundity) of infected individuals. Resistance and tolerance can both increase the fitness of an infected host individual, but resistance does so by necessarily limiting pathogen fitness, whereas tolerance does not.

Resistance and tolerance often incur physiological costs that manifest as reduced host fitness in the absence of disease in comparison to susceptible (i.e., neither resistant nor tolerant) hosts. Such costs can arise because the defense strategy has harmful pleiotropic effects (Parker 1990) or because investment in defense requires allocation of limiting resources and hence trade-off with other life-history traits. Costs of maintaining resistance in the absence of infection have been demonstrated in a wide variety of organisms, including plant (Bergelson and Purrington 1996; Biere and Antonovics 1996; Tian et al. 2003), insect (Boots and Begon 1993; Kraaijeveld and Godfray 1997; Fellowes et al. 1998; Koella and Boëte 2002; Koskela et al. 2002), and bacterial species (Gómez and Buckling 2011). Trade-offs between tolerance to parasitism and other fitness-determining traits have been demonstrated in several plant species (Simms and Triplett 1994; Koskela et al. 2002; but see Carr et al. 2005). Resistance and tolerance can evolve only if the advantages that ensue should infection occur outweigh the costs of maintaining the defenses in the absence of infection, incorporating the probability of pathogen encounter. Costs can therefore be important constraints on the evolutionary dynamics of defenses (Restif and Koella 2004).

Resistance and tolerance traits, even given equivalent direct single-generation effects on host fitness (i.e., similar protective benefits and costs), can have different epidemiological consequences and therefore exhibit fundamentally different evolutionary behaviors. By limiting pathogen growth and reproduction, resistant hosts can reduce further transmission of the pathogen and thus disease prevalence in the population. As resistant hosts increase in frequency relative to susceptible hosts, the fitness advantage of resistant hosts declines along with the decreasing probability of pathogen encounter. Such negative frequency-dependent selection is expected to maintain polymorphism in resistance over a wide range of costs (Boots and Bowers 1999). In contrast, tolerance does not negatively affect pathogen transmission and therefore has a neutral or even positive effect on disease prevalence (Roy and Kirchner 2000; Miller et al. 2006; Boots 2008). As tolerant hosts increase in frequency, disease prevalence also increases, conferring an ever greater relative fitness advantage to tolerant hosts over susceptible hosts. Thus, a positive epidemiological feedback is expected to promote the fixation of tolerance in the absence of other factors influencing selection (Roy and Kirchner 2000). By favoring the spread of the pathogen, tolerant hosts also impose elevated disease burdens on competitors (i.e., “the use of parasites as a biological weapon”; Restif and Koella 2004). In spite of the positive feedback of tolerance, genetic variation in tolerance has been noted in several plant and animal systems (Koskela et al. 2002; Råberg et al. 2007, 2009; Read et al. 2008).

There is growing recognition that the spatial structure of populations can markedly impact the evolution of disease-related traits. Theoretical (Rand et al. 1995; Boots and Sasaki 1999; Haraguchi and Sasaki 2000; Kamo et al. 2007) and empirical studies (Kerr et al. 2006; Boots and Mealor 2007) have shown that pathogens in spatially structured populations tend to evolve lower transmission rate and virulence than in completely mixing populations. Furthermore, in spatially structured populations, localized transmission and host reproduction can select for higher host resistance, in comparison to well-mixed populations (Best et al. 2011). The evolutionary behavior of tolerance may also depend on spatially structured ecological interactions, especially given that tolerance is expected to increase local pathogen prevalence. Previous theoretical studies of tolerance evolution, however, have not accounted for spatial population structure (Roy and Kirchner 2000; Restif and Koella 2004; Best et al. 2008).

Here we consider the evolution of resistance and tolerance with respect to their associated costs using a spatially explicit, individual-based stochastic simulation model. We assume that hosts exist on a regular lattice and that ecological interactions, such as pathogen transmission and host reproduction, can occur either locally between neighboring sites or globally between all sites on the lattice. Using pairwise invasion trials, we show that tolerance has an advantage relative to resistance when invading into a population of susceptible hosts. However, in a spatially structured setting, tolerance has a disadvantage relative to resistance when invading into a resident population containing well-defended resistant hosts. Our results show that spatial genetic structure can restrict the evolution of tolerance and extend our understanding of the relative advantages and costs of these forms of defense and the ease with which tolerance can evolve in host–parasite systems with spatial structure.

## Methods

We consider a regular 100 × 100 toroidal lattice of sites, which may each be empty or occupied by a host individual. Hosts may be of three asexual haploid genotypes, resistant (*R*), tolerant (*T*), or susceptible (*S*). Resistance is defined here as the ability of the host to reduce the growth of the pathogen, thus reducing both the loss of host fecundity due to infection and the pathogen transmission from the infected host, but not the probability of becoming infected (avoidance). Tolerance is defined as the host's ability to offset the negative effects of infection on host fecundity, but without limiting pathogen growth or reproduction and thus without effects on pathogen transmission.

Hosts may be infected by the pathogen and cannot recover from infection. Each host individual has a genotype *G*, which can take the values of *R*, *T*, or *S*, and an infection status *i*, where *i* = 1 if the individual is infected and *i* = 0 if uninfected. Together these characteristics determine the host's fecundity *r_G,i_* and transmission rate β*_G,i_*. Infection reduces host fecundity (*r_G_*,_1_ < *r_G_*,_0_ for all *G*) without affecting mortality. We checked that an otherwise identical model in which infection instead caused higher host mortality yielded qualitatively similar outcomes.

To determine the evolutionary consequences of the different epidemiological consequences of resistance and tolerance, we considered resistance and tolerance traits that confer equivalent direct fitness benefits to infected individuals (*r_R_*,_1_ = *r_T_*,_1_ > *r_S_*,_1_). In uninfected hosts, resistance and tolerance each incur a cost manifested as reduced fecundity of uninfected individuals relative to susceptible individuals (*r_R/T_*,_0_ < *r_S_*,_0_). For convenience, we refer to the cost of defense *c_G_* = 1 −ρ*_G_*, where ρ*_G_* = *r_G_*,_0_/*r_S_*,_0_ expresses the fecundity of uninfected resistant or tolerant individuals relative to uninfected susceptible individuals.

In our model, the fundamental difference between resistance and tolerance is in the pathogen transmission rate from infected individuals. Because resistance limits pathogen growth, the pathogen has lower transmission rate from infected resistant hosts than from infected susceptible hosts (β*_R_*,_1_ < β*_S_*,_1_). Tolerance, in contrast, does not limit pathogen growth; thus, the transmission rate from infected tolerant hosts is equal to that from infected susceptible hosts (β*_T_*,_1_ = β*_S_*,_1_). No pathogen transmission occurs from uninfected individuals.

The model is iterated through synchronous time steps that consist of host reproduction, transmission, and death. First, host individuals reproduce into an adjacent empty site with a probability of success given by their fecundity *r*. Successful reproduction results in the birth of a single uninfected individual with clonally inherited genotype in a neighboring empty site. Next, infection is transmitted from infected to uninfected individuals. In the spatial model, infection is transmitted locally: each uninfected host has a probability per time step of becoming infected by any particular neighbor equal to the transmission rate β from that neighbor; transmission from each infected neighbor occurs independently. In the nonspatial model, infection is transmitted globally: each uninfected host becomes infected with probability per time step equal to the global infection density (i.e., average number of infected individuals per site); modifications to the model by weighting global transmission by the transmission rate β of each individual and by using iterations with global reproductive dispersal of the host as a means to disrupt local spatial structure gave qualitatively similar results. Lastly, individuals are removed by death at a fixed mortality rate μ.

We studied the pairwise invasion of either resistant or tolerant hosts into susceptible populations, and the invasion of tolerant hosts into resistant populations. The complementary case of invasion of resistant hosts into tolerant populations was also examined and yielded outcomes qualitatively identical to the invasion of tolerant hosts into resistant populations (data not shown). In each case, we varied the relative costs of the defense traits to determine the effect of cost on invasion ability. Because the defense traits confer equivalent direct protective benefit, any differences in the relative costs at which resistance and tolerance can invade reflect differences in the overall fitness advantage provided by the defenses, accounting for their divergent epidemiological feedbacks. In trials of the invasion of resistance or tolerance into susceptible populations, we varied the cost of defense *c_R_*_/_*_T_* from 0 to 0.50 in increments of 0.02. In trials of the invasion of tolerance into resistant populations, we varied the cost of resistance *c_R_* between 0 and 0.50 in increments of 0.02, while the cost of tolerance was varied in increments of 0.008 about a given cost of resistance. These particular values were chosen to allow reasonable resolution of cost differences within computational constraints. Invasion conditions were examined under both local and global transmission for each combination of costs.

In each invasion trial, the lattice was initially populated with 1000 host individuals distributed randomly among sites, each with 0.25 probability of being infected. The simulation was run for 1000 time steps to allow the resident population to equilibrate. Invading hosts (50 individuals, equivalent to about 1% of the resident population) were then introduced into random empty sites; infection prevalence among the invading hosts was set equal to that of the resident host population. The simulation was propagated for either 200,000 time steps or until one type of host reached fixation. Invasion dynamics were characterized by observing infection prevalence and the relative frequencies of the host types. Results from three independent replicates were averaged for each parameter combination.

The parameter values that were explored are presented in Table 1. Generally, parameters were calibrated for the persistence of host and pathogen over a broad range of costs of defense and such that there was polymorphism of resistance over a broad range of costs (Antonovics and Thrall 1994; Boots and Bowers 1999) and fixation of tolerance over the same range of costs (Roy and Kirchner 2000).

**Table 1:** Definitions and calibrated values of model parameters

*r*_*S*,0_	Birth rate of uninfected susceptible hosts	0.25
*r*_*R*,0_	Birth rate of uninfected resistant hosts^1^	0.125–0.25
*r*_*T*,0_	Birth rate of uninfected tolerant hosts^1^	0.125–0.25
*r*_*S*,1_	Birth rate of infected susceptible hosts	0.05
*r*_*R*,1_	Birth rate of infected partially resistant hosts	0.95*r*_*R*,0_
*r*_*T*,1_	Birth rate of infected partially tolerant hosts	*r*_*R*,1_
*r*_*R*,1_	Birth rate of infected completely resistant hosts	*r*_*R*,0_
*r*_*T*,1_	Birth rate of infected completely tolerant hosts	*r*_*T*,0_
β_*G*,0_	Transmission rate from uninfected hosts	0
β_*S*,1_	Transmission rate from infected susceptible hosts	0.25
β_*R*,1_	Transmission rate from infected resistant hosts	0.01
β_*T*,1_	Transmission rate from infected tolerant hosts	0.25
ρ*_R_*	Relative fecundity of uninfected resistant hosts (given by *r*_*R*,0_/*r*_*S*,0_)	0.5–1
ρ*_T_*	Relative fecundity of uninfected tolerant hosts (given by *r*_*T*,0_/*r*_*S*,0_)	0.5–1
*c_R_*	Cost of resistance (given by 1 −ρ*_R_*)	0–0.5
*c_T_*	Cost of tolerance (given by 1 −ρ*_T_*)	0–0.5
μ	Host death rate	0.1

1 Values represent the cost of defense, ranging from 0% to 50% reduction in fecundity relative to susceptible hosts.

## Results

**Figure 1:**
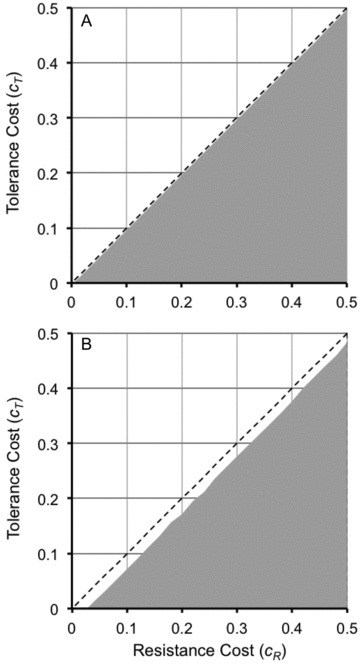
Costs at which tolerance is able to invade a population composed of resistant hosts that have equal fitness loss due to infection. Cost is given as *c_R_*_/_*_T_*, as defined in text. Gray regions show the range of costs over which tolerance can invade. Dashed line indicates equal costs, that is, *c_T_* = *c_R_*. Panel A shows conditions of global transmission, partial defense; Panel B, local transmission, partial defense.

We first explored our model in the nonspatial setting with global pathogen transmission. In this case, tolerance can invade into a population of susceptible hosts with costs up to 0.8% greater than resistance (maximal cost at which tolerance can invade, c_T_* = 0.533; resistance, c_R_* = 0.529), suggesting that tolerance has an advantage relative to resistance during invasion of susceptible populations. Thus, our model replicates the prior result in unstructured susceptible populations that tolerance provides an advantage beyond the direct effects of protection from pathogen-induced fitness loss, in comparison to resistance (Restif and Koella 2004). Tolerance can invade a population of resistant hosts with costs up to those of the resident resistance (Fig. 1A). When the costs of resistance and tolerance are equal, the evolutionary outcome of the invasion of tolerance is stochastic, indicating an absence of selective advantage to either resistance or tolerance.

We next introduced spatial structure to the model by considering local pathogen transmission. Our model exhibits highly clustered distributions of genetically related hosts due to local dispersal. Local pathogen transmission therefore results in kin-structured patterns of disease transmission. Under these conditions, tolerance that provides the same level of protection against disease as resistance can invade into a population of susceptible hosts with a substantially (up to 8%) higher cost than resistance (c_T_* = 0.496; c_R_* = 0.460). However, in spatially structured populations that contain only resistant hosts, tolerance can invade only if it has a lower cost than resistance that provides equivalent direct benefits (Fig. 1B). Over the range of costs from 0 to 0.50 reduction in the fecundity of uninfected hosts, tolerance successfully invaded populations of resistant hosts only when paying at minimum 3% lower cost, relative to the cost of resistance. This finding suggests that hosts with tolerance suffer a fitness disadvantage when competing against resistant hosts (assuming both traits confer equivalent direct benefits).

Hosts with complete resistance and tolerance defenses suffer no fitness loss due to infection (i.e., *r*_*R*,0_ = *r*_*R*,1_ and *r*_*T*,0_ = *r*_*T*,1_). We found that complete resistance and tolerance show different evolutionary behavior than partial defenses in the spatial setting. Complete tolerance can invade a population of susceptible hosts with substantially (up to 7%) higher cost than complete resistance (c_T_* = 0.496; c_R_* = 0.462), with the difference in costs comparable to that observed in the case of partial defense. Thus, complete tolerance has an advantage relative to complete resistance when invading susceptible populations. However, completely tolerant hosts can invade a population of completely resistant hosts with costs up to those of the resident resistant hosts, indicating that complete tolerance, unlike partial tolerance, suffers no disadvantage when competing with resistance that confers equivalent direct protective benefits.

## Discussion

Resistance and tolerance each mitigate the harm caused by infectious diseases, but do so in ways that differ fundamentally in how they affect pathogen persistence and prevalence. In 2004, Restif and Koella suggested that tolerance favors the spread of pathogen and thus creates a harsh pathogenic environment for susceptible competitors. This effect of tolerance on competition among hosts represents an additional benefit that can allow tolerance to evolve with higher cost than resistance. Our results suggest that, on the one hand, this additional benefit of tolerance relative to resistance occurs when the defense arises in a population of susceptible host individuals that suffer relatively greater harm, in terms of fitness, due to disease. On the other hand, when tolerance is introduced into populations composed of equally well-defended resistant hosts, the increased pathogen burden does not harm the resistant hosts any more than it harms the tolerant hosts themselves; thus, no additional competitive advantage is afforded to tolerance. Our results therefore suggest that the presence of resistance can potentially limit the evolution of tolerance.

Many natural systems exhibit spatial genetic structure over short distances due to limited dispersal of gametes or offspring (Loiselle et al. 1995; Shapcott 1995). In our model, local reproduction generates a clustered distribution of genetically related hosts, with each tolerant host likely existing within a cluster composed of other tolerant hosts. Under conditions of local pathogen transmission, such clusters of tolerant hosts experience locally elevated prevalence of infection. The average partially tolerant host therefore suffers greater fitness loss due to disease than the average resistant host, because the latter tends to exist within clusters with relatively lower infection prevalence (perhaps analogous to an Allee effect where the benefit of group size depends entirely upon the phenotype of its members). When tolerance is introduced into a population composed of only susceptible hosts (i.e., resistant hosts are absent), the disease burden of locally elevated infection prevalence is offset by the competitive advantage gained from imposing disease upon the more severely harmed susceptible individuals. Tolerance can therefore invade with higher cost than resistance into a population of only susceptible hosts. However, when partial tolerance is introduced into a population of equally well-defended resistant hosts, the burden of infection prevalence is not counterbalanced by any benefit arising from the imposition of disease on the resistant competitors. In this setting, partial tolerance can invade only with costs lower than those associated with resistance. Thus, our results suggest that spatial genetic structure can represent a major restriction to the evolution of partial tolerance, especially in the context of resistance. The magnitude of the disadvantage to tolerance relative to resistance is expected to depend on the degree of spatial genetic structure within the host population, implying that tolerance is less likely to evolve in systems with low host dispersal.

Unlike hosts that have partial defenses, hosts with complete defenses, by definition, suffer no fitness loss due to infection. Completely tolerant hosts therefore experience no self-imposed fitness burden due to locally elevated infection prevalence, yet, still benefit from imposing disease-induced harm upon susceptible competitors. Consequently, complete tolerance can invade susceptible populations with costs greater than those of complete resistance and can invade resistant populations with costs up to those of resistance.

While epidemiological feedbacks have been recognized as an important driver of the evolution of defenses (Boots et al. 2009), previous theoretical studies have not accounted for the spatial structure of host populations. Using a nonspatial model that formally defines resistance as a reduction in pathogen transmission, Roy and Kirchner (2000) predicted that tolerance (defined here as reduced fecundity loss due to infection) will generally evolve to fixation. Restif and Koella (2004) showed that mixed strategies with investment in both resistance and tolerance can be evolutionarily stable, but again in the nonspatial setting. The nonspatial model of Best et al. (2008) showed that trade-offs between resistance and tolerance can maintain genetic variation in both traits. Our spatially explicit model reveals that local epidemiological feedbacks in viscous populations can constrain the evolution of tolerance. This is consistent with recent theoretical studies of defense evolution in spatial settings that have indicated that local interactions favor the evolution of resistance strategies (Best et al. 2011), while tolerance can be less favored (Débarre et al. 2012). Because our model defines both resistance and tolerance directly in terms of their effects on pathogen transmission, we were able to determine the evolutionary consequences of their divergent epidemiological effects.

Given that disease resistance is widespread in natural populations, the introduction of tolerance by either mutation or migration probably often occurs in populations that contain resistant hosts. Our findings suggest that barriers to the evolution of tolerance within spatial settings may be greater in the presence of resistance. This may contribute some understanding to the puzzling observations of variation in tolerance within and among populations (Koskela et al. 2002; Råberg et al. 2007, 2009), despite the theorized ease with which tolerance is expected to evolve to fixation (Roy and Kirchner 2000; but see Best et al. 2008). The frequency of resistant hosts is likely to vary within and among populations due to ecological, coevolutionary, and historical factors, and this would correspondingly generate temporally and spatially varying selective pressures upon tolerance. Our results support the idea that tolerance should be less common in populations where resistance is present in high frequency. However, care must be taken in experimental assessment, as trade-offs between tolerance and resistance might produce a qualitatively similar pattern. In future theoretical studies, it will be important to explore the combined interactions and dynamics of all three types of hosts (i.e., susceptible, resistant, and tolerant) in order to generate predictions that can be tested against empirical observations.

The main driver of the relative fitness of resistant and tolerant genotypes is the relative amount of pathogen transmission from infected hosts. Mixed strategies of defense are therefore likely to exhibit distinct evolutionary dynamics (Restif and Koella 2004). For given levels of direct protection and cost, our results suggest that any shift from tolerance toward resistance will be selectively favored because it will reduce the negative fitness effects associated with elevated infection prevalence in its neighborhood. In many situations, it is therefore likely that investment in tolerance alone is an evolutionarily unstable strategy, and this may provide some additional understanding of observed variation in tolerance within populations (Koskela et al. 2002). While this prediction is consistent with recent studies showing that higher investment in resistance is favored under conditions of local transmission and host reproduction (Best et al. 2011; Débarre et al. 2012), the evolution of mixed defense strategies in a spatial setting remains to be investigated. In addition, future models should consider the possibility of coevolution of hosts and parasites, which was neglected here for the sake of simplicity, but is thought to be an important driver of evolutionary dynamics in many natural systems. The incorporation of space into theoretical models is an exciting approach that promises to enrich our understanding of host–pathogen evolutionary dynamics.

## References

[b1] Antonovics J, Thrall PH (1994). The cost of resistance and the maintenance of genetic polymorphism in host-pathogen systems. Proc. R. Soc. B Biol. Sci.

[b2] Bergelson J, Purrington CB (1996). Surveying patterns in the cost of resistance in plants. Am. Nat.

[b4] Best A, White A, Boots M (2008). Maintenance of host variation in tolerance to pathogens and parasites. Proc. Natl. Acad. Sci.

[b3] Best A, Webb S, White A, Boots M (2011). Host resistance and coevolution in spatially structured populations. Proc. R. Soc. B Biol. Sci.

[b5] Biere A, Antonovics J (1996). Sex-specific costs of resistance to the fungal pathogen *Ustilago violacea**Microbotryum violaceum*) in *Silene alba*. Evolution.

[b6] Boots M (2008). Fight or learn to live with the consequences?. Trends Ecol. Evol.

[b7] Boots M, Begon M (1993). Trade-offs with resistance to a *Granulosis* virus in the Indian meal moth, examined by a laboratory evolution experiment. Funct. Ecol.

[b9] Boots M, Bowers RG (1999). Three mechanisms of host resistance to microparasites—avoidance, recovery and tolerance—show different evolutionary dynamics. J. Theor. Biol.

[b10] Boots M, Mealor M (2007). Local interactions select for lower pathogen infectivity. Science.

[b11] Boots M, Sasaki A (1999). “Small worlds” and the evolution of virulence: infection occurs locally and at a distance. Proc. R. Soc. B Biol. Sci.

[b8] Boots M, Best A, Miller MR, White A (2009). The role of ecological feedbacks in the evolution of host defence: what does theory tell us?. Phil. Trans. R. Soc. B.

[b12] Carr DE, Murphy JF, Eubanks MD (2005). Genetic variation and covariation for resistance and tolerance to cucumber mosaic virus in *Mimulus guttatus* (Phrymaceae): a test for costs and constraints. Heredity.

[b13] Débarre F, Lion S, van Baalen M, Gandon S (2012). Evolution of host life-history traits in a spatially structured host-parasite system. Am. Nat.

[b14] Fellowes MDE, Kraaijeveld AR, Godfray HCJ (1998). Trade-off associated with selection for increased ability to resist parasitoid attack in *Drosophila melanogaster*. Proc. R. Soc. B Biol. Sci.

[b15] Gómez P, Buckling A (2011). Bacteria-phage antagonistic coevolution in soil. Science.

[b16] Haraguchi Y, Sasaki A (2000). The evolution of parasite virulence and transmission rate in a spatially structured population. J. Theor. Biol.

[b17] Kamo M, Sasaki A, Boots M (2007). The role of trade-off shapes in the evolution of parasites in spatial host populations: an approximate analytical approach. J. Theor. Biol.

[b18] Kerr B, Neuhauser C, Bohannan BJM, Dean AM (2006). Local migration promotes competitive restraint in a host–pathogen “tragedy of the commons.”. Nature.

[b19] Koella JC, Boëte C (2002). A genetic correlation between age at pupation and melanization immune response of the yellow fever mosquito *Aedes Aegypti*. Evolution.

[b20] Koskela T, Puustinen S, Salonen V, Mutikainen P (2002). Resistance and tolerance in a host plant-holoparasitic plant interaction: genetic variation and costs. Evolution.

[b21] Kraaijeveld AR, Godfray HCJ (1997). Trade-off between parasitoid resistance and larval competitive ability in *Drosophila melanogaster*. Nature.

[b22] Loiselle BA, Sork VL, Nason J, Graham C (1995). Spatial genetic structure of a tropical understory shrub, *Psychotria officinalis* (Rubiaceae). Am. J. Bot.

[b23] Miller MR, White A, Boots M (2005). The evolution of host resistance: tolerance and control as distinct strategies. J. Theor. Biol.

[b24] Miller MR, White A, Boots M (2006). The evolution of parasites in response to tolerance in their hosts: the good, the bad, and apparent commensalism. Evolution.

[b25] Parker MA (1990). The pleiotropy theory for polymorphism of disease resistance genes in plants. Evolution.

[b26] Price PW (1980). Evolutionary biology of parasites.

[b28] Råberg L, Sim D, Read AF (2007). Disentangling genetic variation for resistance and tolerance to infectious diseases in animals. Science.

[b27] Råberg L, Graham AL, Read AF (2009). Decomposing health: tolerance and resistance to parasites in animals. Phil. Trans. R. Soc. B Biol. Sci.

[b29] Rand DA, Keeling M, Wilson HB (1995). Invasion, stability and evolution to criticality in spatially extended, artificial host-pathogen ecologies. Proc. R. Soc. B Biol. Sci.

[b30] Read AF, Graham AL, Råberg L (2008). Animal defenses against infectious agents: is damage control more important than pathogen control. PLoS Biol.

[b31] Restif O, Koella JC (2004). Concurrent evolution of resistance and tolerance to pathogens. Am. Nat.

[b32] Roy BA, Kirchner JW (2000). Evolutionary dynamics of pathogen resistance and tolerance. Evolution.

[b33] Shapcott A (1995). The spatial genetic structure in natural populations of the Australian temperate rainforest tree *Atherosperma moschatum* (Labill.) (Monimiaceae). Heredity.

[b34] Simms EL, Triplett J (1994). Costs and benefits of plant responses to disease: resistance and tolerance. Evolution.

[b35] Tian D, Traw MB, Chen JQ, Kreitman M, Bergelson J (2003). Fitness costs of R-gene-mediated resistance in *Arabidopsis thaliana*. Nature.

